# Living life in limbo: experiences of healthcare professionals during the HCPC fitness to practice investigation process in the UK

**DOI:** 10.1186/s12913-021-06785-7

**Published:** 2021-08-19

**Authors:** Jill Maben, Linda Hoinville, Dawn Querstret, Cath Taylor, Magdalena Zasada, Ruth Abrams

**Affiliations:** 1 School of Health Sciences, Faculty of Health and Medical Sciences, University of Surrey, Kate Granger Building, Priestley Road, Surrey Research Park, GU2 7YH Guildford, UK; 2 Department of Psychology and Pedagogic Science, Faculty of Sport, Health and Applied Science, St Mary’s University, Waldegrave Road, Twickenham, TW1 4SX London, UK

**Keywords:** Fitness to practice, Healthcare professionals, Emotional impact, Staff well-being

## Abstract

**Background:**

It is the responsibility of healthcare regulators to ensure healthcare professionals remain fit for practice in healthcare settings. If there are concerns about an individual healthcare professional they may undergo a fitness to practice investigation. This process is known to be hugely stressful for doctors and social workers, but little is known about the impact of this experience on other professions. This study explores the experiences of registrants going through the process of being reported to the UK’s Health and Care Professions Council (HCPC) and attending fitness to practice (FTP) hearings. We discuss the implications of this process on registrants’ wellbeing and, from our findings, present recommendations based on registrants experiences. In doing so we articulate the structural processes of the HCPC FTP process and the impact this has on individuals.

**Methods:**

This study uses semi-structured interviews and framework analysis to explore the experiences of 15 registrants who had completed the FTP process. Participants were sampled for maximum variation and were selected to reflect the range of possible processes and outcomes through the FTP process.

**Results:**

The psychological impact of undergoing a FTP process was significant for the majority of participants. Their stories described influences on their wellbeing at both a macro (institutional/organisational) and micro (individual) level. A lack of information, long length of time for the process and poor support avenues were macro factors impacting on the ability of registrants to cope with their experiences (theme 1). These macro factors led to feelings of powerlessness, vulnerability and threat of ruin for many registrants (theme 2). Suggested improvements (theme 3) included better psychological support (e.g. signposting or provision); proportional processes to the incident (e.g. mediation instead of hearings); and taking context into account.

**Conclusions:**

Findings suggest that improvements to both the structure and conduct of the FTP process are warranted. Implementation of better signposting for support both during and after a FTP process may improve psychological wellbeing. There may also be value in considering alternative ways of organising the FTP process to enable greater consideration of and flexibility for registrants’ context and how they are investigated.

**Supplementary Information:**

The online version contains supplementary material available at 10.1186/s12913-021-06785-7.

## Background

Healthcare systems around the world are coming under both increasing pressure and scrutiny. Evolving healthcare policies, resource constraints and societal needs such as patient multi-morbidities mean that demands on healthcare professionals are growing. A significant demand comes from changes to societal expectations of healthcare professions roles. These roles are constantly adapting to meet societal needs without necessarily preparing professionals for ever increasing changes and expectations. One example of this may be the role of paramedics, who now find themselves in situations that require care provision that is above and beyond clinical duties including, *“psychosocial support, conflict management, or to intervene in quasi-legal situations (for example, attending at an injury involving domestic abuse)*” [1]. This may create situations where standard clinical assessment combined with contextual factors make straight-forward decision-making more complex and ambiguous. This is particularly pertinent in healthcare systems that encourage collaborative, distributed and relational decision-making which, as Goodwin suggests [2], require a broader definition of accountability to reflect contextual factors and the diffuse nature of decision making. Furthermore, Waring [3] has highlighted that although knowledge about patient safety is socially constructed within clinical settings, incident reporting and risk management activities de-contextualise and reconstruct it in a way that conceals latent factors and indicates individual culpability. The need to account for context in the construction of safety narratives has been brought to the fore by the Bawa-Garba case, involving the death of a child for which Dr Bawa-Garba was convicted of gross negligence manslaughter. There were systemic issues at the hospital including staff shortages, IT systems failures and lack of accessibility of data at the bedside. This case has led to subsequent calls for consideration of corporate responsibility to take account of the contextual influences on healthcare and patient safety [4]. Despite this and depending on the care outcome, staff may find themselves subject to patient complaints and regulatory processes, to justify decisions they make in their practice with little account of the contextual factors influencing those decisions or actions. Indeed, the last decade has seen formal patient complaints double [5].

Worldwide, the conduct and professional practice of healthcare professionals is governed by healthcare regulators who set and monitor standards of qualification and practice in order to ensure safe practice [6]. Different countries have different health professional regulation models [7]. In the UK, investigation of healthcare professional practice can occur through either self-referral or through complaints or concerns being raised by a third party such as a member of the public/patient, or a healthcare organisation. If a regulator receives an allegation or if a healthcare professional self-refers, regulators are legally obliged to investigate it as part of a ‘Fitness to practice’ process (i.e. a conduct, competence or ethics investigation) [8]. Whilst this process may take into account a patient’s experience (if relevant), it focuses specifically on any potential impaired fitness to practice (FTP) of the healthcare professional [9]. A healthcare professional may find themselves temporarily suspended from their practice depending on the case to answer, or they remain on the healthcare professional register throughout the investigation [8].

In the UK there are currently 15 professions^1^ that are regulated by the world’s largest regulator of healthcare professionals, the Health Care Professions Council [10]. The HCPC hold statutory power to suspend the practice of a healthcare professional and/or remove them from the register if the case under investigation deems it appropriate. Their investigative process is designed to enable them, “to take appropriate action to protect the public from those who are not fit to practice either at all or on an unrestricted basis” [11].

Going through the FTP process is a very stressful time for healthcare professionals. Indeed, a systematic review of literature exploring coping with medical errors, concluded that the process of investigation may produce a psychological response in the healthcare professional such as shame, guilt and/or anxiety [12]. Depression and suicidal ideation may be experienced, along with feelings of distrust and detachment towards patients [13–19]. An independent report commissioned by the General Medical Council (GMC) into the impact of FTP processes on doctors indicated that the process itself may exacerbate underlying mental health disorders such as depression, bipolar and personality disorders [19]. Extant research indicates that a healthcare professional’s ability to cope may be significantly reduced due to the culture of medical training, and that workplace support is often insufficient [12, 19]. Enhancing avenues for support, as well as treating those under investigation as *innocent until proven guilty* were key recommendations outlined in the GMC report [19]. Access to workplace support may come in a number of forms such as specific training for management or Human Resource departments to support those going through investigation and potential suspension [20].

FTP processes such as those conducted by the GMC, have been described as depersonalising, dehumanising, accusatory, limited in communication, fraught with legal jargon and unacceptably lengthy [13, 16, 19]. Internationally, Biggar et al. [6], in their Australian study into making healthcare professional regulation processes more humane, indicated that fairness, transparency, communication, timeliness and empathic contact are factors that may have a positive impact on healthcare professionals under investigation.

A large majority of research into the impact of the FTP process on healthcare professionals has been explored in relation to UK Doctors [13, 16, 19] or social workers [8]. Little is known about how these experiences translate across different professions who are regulated by different professional bodies and may experience different processes. This study therefore aimed to better understand the experiences and challenges of registrants being reported to the HCPC and attending FTP hearings; and to identify how the HCPC can better support registrants when experiencing FTP processes. To our knowledge this is the first study to explore the experiences of registrants going through the process of being reported to the HCPC and attending FTP hearings in the UK.

## Methods

### Participant recruitment and sampling

**Fig. 1:**
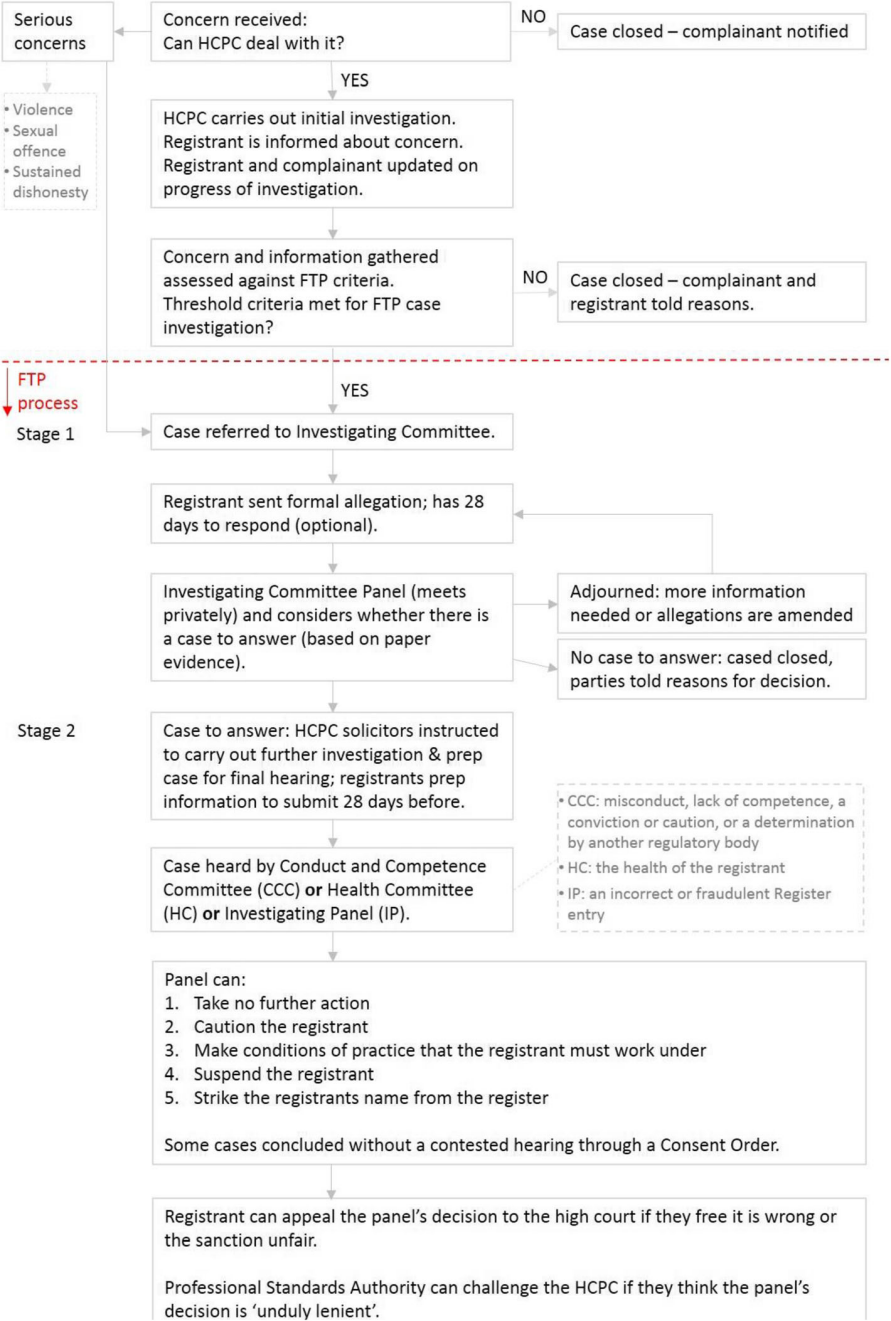
FTP Flowchart

This study was commissioned by the HCPC and explores the experiences of 15 registrants who had completed the FTP process. The sampling included those who had been through the process in the past 12 months, in theory, yet we discovered that because of the lengthy processes, HCPC registrants could have active cases in the last 12 months that started several years previously. Participants were purposively sampled for maximum variation in relation to professional group; range of possible stages and panels (‘Investigating Committee’ stage and/or to ‘Panel Hearing’ stage; i.e. they had either been through ‘Health’ panel or ‘Conduct and Competence’ panel (and in one case both); and range of outcomes (No case to answer, Caution, Conditions of Practice, Suspension) (see Fig. 1; Table 1). Individuals who are seen by the Investigating Panel for fraudulent registrations or who had been struck off the register were excluded from this study because we wanted to understand recent experiences and had no way of making contact with those no longer registered with the HCPC. A total of 91 potential participants were identified and initially contacted by email by the HCPC. If willing to participate they contacted the research team directly by email and were provided with the participant information sheet detailing the research aims, the funder and the researcher’s details. Participants’ details were kept confidential and not shared with the HCPC. Twenty-three participants contacted researchers to express an interest in taking part; 18 agreed to interview and three dropped out. Two did not want to re-live their experiences and another did not respond to emails to set up an interview. Table 1 explains the terminology used in relation to stages, panels and outcomes. Figure 1 provides an overview of the FTP process.

**Table 1:** Terminology

Term	Description
Investigating Committee stage	The point at which all evidence is reviewed together and a decision is made (by the committee) about whether there is a case to answer in respect of the allegation against the registrant.
Panel Hearing stage	The point at which the individual’s circumstances are considered and they are able to present their case in relation to evidence under review.
Health panel	A panel established to review evidence where ill health has been cited as the reason for impairment to practice.
Conduct and Competence panel	A panel established to review evidence regarding misconduct, lack of competence, a conviction or caution, or a determination by another regulatory body.
No case to answer	No further action is required or taken.
Caution	A caution order can last between 1–5 years and appears next to a registrant’s name on the register.
Conditions of Practice	Restrictions are placed on the registrants. They may be required to work under supervision or be asked to undertake additional training.
Suspension	The registrant is no longer able to practice. This can last for one year, or they can be struck off the register permenantly.

Adapted from HCPC Annual Report, 2010 [9]

Ethical approval was obtained from the University of Surrey Research Ethics Committee (ref: UEC/2019/041/FHMS) prior to commencing the work. We have been fully compliant with data protection (GDPR) and have received written informed consent from all participants for participation in interviews, and for the use of their data for publication. Quotes are anonymised with pseudonyms to protect identities.

### Data collection and analysis

Given the sensitive nature of the experience, interviews were all held face-to-face and were conducted by JM, LH and DQ. These researchers were motivated to gather participants’ experiences and to make changes to the HCPC processes as needed. They had no prior relationships with any of the participants. The topic guide for interviews was informed by the literature and the objectives of this research. Topics included participant background, prompts to elicit their FTP ‘story’ and information received from HCPC, experience of the process, including relationships with staff, the investigation and hearing processes, how registrants coped with the process, support received and from whom, overall questions about the best and worst parts of the FTP process and asking what HCPC could do differently to make the process better. The interview topic guide was developed for this study and is provided as Supplementary file 1. Interviews were semi-structured to enable other non-anticipated topics or issues to emerge from participants and lasted between 60–90 minutes. Interviews were undertaken in settings chosen by the participants and included confidential spaces such as their own homes or the researcher’s office. One interviewee invited their husband to be present, and three interviews has two researchers present to support a rigorous process. All other interviews had only the interviewee and the researcher. A narrative approach was used and an initial question asked interviewees to ‘tell their story” wherever they would like to start. Interviews were audio-recorded with permission and transcribed verbatim for analysis. Data saturation was achieved with fifteen interviews. Transcripts were returned to participants for verification and to check if they were happy for us to use quotations from the data they had provided. Data were analysed using the Framework analysis method to categorise the data thematically [21]. This included investigation of patterns within and between individual participants through familiarisation and coding. Four members of the research team (JM, MZ, LH, CT) were involved in coding and analysing the data (Table 2). An analytical framework was then generated (Supplementary file 2) and applied to support the development of themes to answer the study aim and objectives. A write up of the findings was provided to participants for verification and feedback. Table 2 provides further details on the analytical process.

**Table 2:** Framework analysis stages

Stage of analysis process	Analysis procedures
Stage 1: Transcription	Audio recordings were transcribed verbatim.
Stage 2: Familiarisation	LH reviewed all of the transcripts to develop ideas for the initial codes. JM, CT, MZ read a sample of transcripts.
Stage 3: Coding	LH created an initial coding structure based on the interview guide and the initial review of all transcripts. Three transcripts were selected to illustrate a range of different participant experiences. Initial codes were applied to each of these transcripts by LH with one other member of the research team for each transcript (JM, CT, MZ).
Stage 4: Developing a working analytical framework	The research team met to discuss the initial codes. The outcome of these discussions generated a working analytical framework of codes grouped into categories.
Stage 5: Applying the analytical framework	The analytical framework was used to identify sections of the transcripts that were thought to be relevant to each of the codes. LH cut and pasted from the transcripts into an excel spreadsheet.
Stage 6: Charting into the framework matrix	LH reviewed this spreadsheet to summarise the data for each participant which was re-organised to identify themes. The emerging themes were discussed with JM.
Stage 7: Interpreting the data	This summary of the data by themes was used together with the extracted sections of the transcripts to draft the findings section of the report which was then discussed with the research team, resulting in themes being re-ordered.

### Findings

The 15 health care professionals who participated worked in a range of healthcare professions (see Table 3). Participants had experienced a range of different stages of the FTP process which lasted between one and four years (see Table 4). Four had their case discussed at the investigating committee panel (ICP), who determined that there was no case to answer, one of these attended two interim hearings. The remaining eleven were referred by the ICP to the Conduct and Competence Committee (CCC) or health panels (see Fig. 1. Flowchart of the FTP process). Ten of these attended at least one hearing. These participants experienced a wide range of final outcomes (Table 4).

**Table 3:** Participant characteristics

Characteristic	Number
Sex	
Female	8
Male	7
Profession	
Art therapist	2
Biomedical scientist	1
Practitioner Psychologist	1
Clinical Scientist	1
Dietician	1
Operating department practitioner	3
Paramedic	2
Radiographer	1
Social worker	3
Time in profession (years)	
1-9	3
10-19	4
20-29	7
30+	1
Age (years)	
30-39	3
40-49	3
50-59	7
60+	2

**Table 4:** Summary of participant’s experience of the FTP process

Pseudonym-ID	Referred by	Referred for	Panel referred to^**a**^	Process length (yrs)	Hearings attended	Outcome^**b**^
Samuel-01	Employer	Competence	CCC	2.5	Final, review	COP
Steven-02	Employer	Conduct	CCC	1	None	NFA
Amy-03	Self	Conduct	CCC	2.5	Final	NFA
Susan-05	Employer	Competence	CCC & Health	4	2 Final	COP
Lucy-06	Self	Conduct	ICP	2.5	None	NCTA
Laurence-07	Employer	Conduct	Health	3	Multiple	Suspension
Isabel-08	Employer	Conduct	CCC	2	Interim, final, review	COP
Robert-10	Employer	Conduct	CCC	2	Interim, final	Suspension
Esther-11	Service user	Conduct	ICP	2	None	NCTA
Sophie-12	Service user	Conduct	CCC	3	Final	Caution
Donald-13	Self	Mental health	ICP	1.5	None	NCTA
Sandra-14	Employer	Conduct	CCC	2	Final	NFA
Alex-15	Employer	Competence	CCC	3.5	Final	NFA
Ethan-16	Self	Conduct	CCC	1	Final	Caution
Eleanor-18	Self & Employer	Competence	ICP	2	2 Interim	NCTA

^a^*CCC* conduct and competence, *ICP* investigating committee panel

^b^*NFA* no further action, *COP* conditions of practice, *NCTA* no case to answer

Overall, findings from this study indicate that the majority of participants found the FTP process very difficult, particularly in relation to the psychological impacts on them. The psychological impact on registrants is explained by both macro and micro level impacts of the process. Theme one (macro level) outlines the way in which the institutional mechanisms of the HCPC process, including length of time taken, contributed to the uncertainty and feelings of suspense, which was very difficult to navigate and tolerate for registrants. Theme two (micro level) outlines the ways in which feelings of powerlessness and vulnerability surfaced and why they had such impact on registrants. Whilst there were a range of experiences including both positive and negative, all participants reported that going through the process had a negative impact on their psychological wellbeing and of some also on the psychological well-being of their family. In particular, registrants reported that the process increased feelings of anxiety and shame, and in some cases suicidal ideation. Theme three outlines participants’ coping strategies and suggestions for improving the process, including the provision of additional support and alternative ways of organising the process. Whilst most participants discussed the process negatively, there were a few who suggested that aspects of the HCPC process were balanced, fair and objective.

#### Theme One: A Kafkaesque world: Obfuscation through institutional mechanisms

Participants described the world of HCPC as akin to one of fiction or make-believe. Investigation stages were referred to as surreal and foreign, punctuated by paperwork and states of perplexity:

“*I felt that I was in some sort of process that felt like Alice in Wonderland… (yet) I didn’t believe that I’d done anything wrong.*” (*Susan-05*).

“*You enter a foreign world that feels very precarious, and there’s land mines everywhere. You never feel you’ve got quite enough knowledge to help you navigate where the landmines are or aren’t… And it’s just like you’re dealing with a machine, it feels quite Kafkaesque.*” (*Amy-03*).

Exacerbating this sense of other-worldliness was the length of time taken for the investigation. Over time many participants described their experience of the FTP process with a sense of disbelief. The majority of participants were under investigation for at least two years (Table 4). This period of time was described by some as holding a lot of uncertainty and keeping a registrant in a state of enduring purgatory and limbo, with a sense that something very bad could happen to them at any time:

*“It’s a long time to be in purgatory. Particularly for people with health conditions.”* (*Samuel-01*).

*“Three and a half years is a long time to not know what’s going to happen. Cos there’s the Damocles sword hanging above your head and you don’t know where to go, you can’t make plans at all.”* (*Isabel-08*).

This lack of information about progress combined with the time taken, led participants to feel in a state of suspension and cast adrift, with little feedback:

“*The whole thing was so long and drawn out, and there was no comment about any of this stuff I was sending them, so I just felt like I was … what’s that film, Gravity is it? – I felt like I was on some long string, you know, floating about the universe.*” (*Lucy-06*).

Participants described the effects this had on them with many experiencing high stress levels and not being able to plan for the future:

*“And one’s life is in sort of suspension at that time, it’s seriously seriously difficult… it’s very difficult to apply for jobs, it’s difficult to sort of plan your finances for the year…I might not have a job in a year, I might be… stripped of my professional qualifications, you can’t plan sort of financially to do things – that was very difficult.” (Amy-03)*.

*“They took forever and a day to decide whether this was going to go to their fitness to practise or not. And I’m still hanging on and… when you’re actually the subject of that investigation a week seems like a year. And when potentially it’s your registration, and with that your career that’s on the line–a career that I adore…the stress levels were just astronomical.” (Steven-02)*.

A commonly held perception was that the main aim of the HCPC was to strike registrants off the professional register, rather than provide them with opportunities for learning and support; *“The impression that the HCPC give is that they are there to strike people off” (Donald-13)*, and that HCPC treated registrants as: *“guilty till proven innocent” (Laurence-07)*. Feelings of being dominated by the process were much more likely to occur than feelings of empowerment or learning, shaping the way in which participants felt able to respond or engage. Indeed, several participants talked about the inflexibility they experienced when engaging with the HCPC FTP process:

*“[It’s] a process they can follow step by step by step…here’s our train tracks, we’ve got two wheels that can’t move off of those train tracks … we are not a car or a bike that can move in different directions.” (Steven-02)*.

Other participants spoke about the way they felt treated by HCPC. Oftentimes this was perceived as being treated with disinterest or disregard. One way in which this manifested for participants included experiencing a delay in communication:

“*Whenever I left a message it could be a week before someone got back to me… I was absolutely flabbergasted that they could take so long… it was inexcusable. If I was to do that in my practice now I’d be straight back in front of the HCPC for Fitness to Practice.” (Donald-13)*.

Another manifestation included a lack of information provided by HCPC about the process, timelines and their progress during the investigation, which was a major problem for almost all participants:

*“They changed the case manager a few times as well which didn’t help so there was no consistency there…And there’s very little contact at all, and most of it was me chasing…emails didn’t get answered, phone calls wouldn’t be returned, and it was just infuriating… it would take weeks and months to get responses.” (Samuel-01)*.

Some felt that throughout the process, the HCPC was a faceless, untouchable force, one that could not be challenged. This at times created a feeling of helplessness in participants:

*“They’re not answerable to anybody at all… the impression we got is that they are God and they will do whatever they want to do, there’s nothing you can do about it.” (Sophie’s husband-12)*.

“Everyone is s**t scared of the HCPC.” (Susan-05).

Participants talked about feeling highly vulnerable and on show during the process, with little to no anonymity protecting their professional reputation: *‘My one thing – the registrant should be anonymous” (Esther-11)*. For healthcare professionals, their very identity may be strongly linked to their profession and professionalism [20], rectifying the public nature of investigation to ensure confidentiality and anonymity for registrants was an important issue for several participants, and highlighted as a potential safety issue by one registrant:

*“They expose you on the website for everybody to see. If you’re not suspended you shouldn’t be exposed on the HCPC website.” (Alex-15)*.

*“Just typing in my name onto the HCPC website and getting that kind of information…is the worst thing.” (Robert-10).*

In contrast, some found the process to be objective and those who found the process reassuring were more likely to have self-referred, which one participant being motivated by a desire to: *“have some sensibility in this judgement, there could be a bigger lens through which to look at it” (Amy-03).*

In summary, registrants described the various ways that HCPC institutional structures and processes served to obscure a registrant’s ability to glean insight into how they can best navigate the FTP process. In this theme, we have highlighted the ways in which participants experienced institutional mechanisms as blocks to their understanding of the FTP process (e.g. length of time taken, one-way communication, suspense). Many participants felt positioned as ‘guilty’ from the outset. In the following theme we outline the consequences of these obscuring institutional mechanisms as experienced at a micro level for participants.

#### Theme Two: Powerlessness, vulnerability and the threat of ruin

At an interpersonal level, about half of participants felt that the absence of face-to-face contact meant that they were unable to voice their side of the story. This appeared to foster feelings of powerlessness and invisibility:

*“I didn’t feel they wanted to know the real ins and outs of what went on from my perspective. There was no face to face contact at all. It was all electronic or written…I wanted them to see and hear me as a person, not as a written word.”* (*Steven-02*).

*“I phoned various different times and I’ve always got the kind of like ‘We don’t really want to have any kind of conversation with you, it’s all in the letter’.” (Robert-10)*.

Other times, it was suggested that HCPC were not interested in taking into account the personal contexts of participants, including ill-health prior to the investigation that was later considered relevant at the hearing:

*“My barrister wanted to go down a health route…they refused…But they were quite sympathetic to the health issues during the hearing…in fact they’ve stated directly that it was likely caused because of my health problems.” (Samuel-01)*.

Some participants found the lack of empathy and understanding exhibited by the HCPC towards their position particularly distressing:

*“They have no … just no concept of what we go through… I don’t think I’ve been treated with any respect. I think there’s been a complete lack of sensitivity.” (Alex-15)*.

*“[I was] barely treated like a human to be perfectly honest.” (Samuel-01)*.

All participants reported that the stress of going through the process had impacted negatively on their mental wellbeing. Most mentioned anxiety and constant worry, several described experiencing severe mental health issues including suicidal thoughts:

*“I couldn’t get a job, and I got really really depressed, tried to slash my wrists, because my house was going down the pan, I couldn’t afford the mortgage because I couldn’t get a job. And they were stopping me getting [respondent’s profession] jobs, so I didn’t have anything else that I could do, because that’s all I’ve done all my life.” (Lawrence- 07)*.

“*I had frequent suicidal thoughts*.” (*Isabel-08*).

Residual anxiety and PTSD were mentioned by a number of participants and for some, receiving the referral paperwork was particularly traumatic and triggering:

“*The experience with the HCPC has been so bad that I have major trigger points and flashbacks to the experiences… as soon as I see HCPC anywhere I cower.” (Alex-15)*.

“*I had nightmares about the feelings (I had)… I’ve never felt what I felt (when I was) sat on those stairs – it was like taking a bullet, reading this bundle of information.” (Eleanor-18)*.

As well as living with extreme stress or prolonged anxiety as a result of the process, participants also experienced material or psychological loss. A loss of employment, livelihood and working relationships were all discussed as points of significant distress to participants:

*“I was unemployed, I was unable to gain employment in my profession – I had to take a massive salary drop…I’d just gone from being on a professional salary to now having no money…My whole life stopped – my professional status, my job, my income – I’d lost everything.” (Eleanor-18)*.

*“I basically lost my job of 22 years… it’s almost like a divorce… or a bereavement… I went from a position where I was like an older statesman of the department… and that’s a loss for me as well as for them.” (Robert-10)*.

In addition to the financial losses several participants described negative impacts on relationships with their colleagues:

“*This process is a bully’s charter…those undergoing this process have pariah status within their departments… Everyone knew and started to treat me like the proverbial injured bird – I felt I was being pecked to death.” (Susan-05)*.

“*People ostracising me…you’ve got colleagues that are throwing as many bananas on the floor as they can, antagonising you as much as they can.*” *(Steven-02)*.

Whilst the process had a negative impact on the wellbeing of all registrants’, gaining closure from HCPC in the form of the final letter had a positive impact on several registrants:

“*So receiving that final letter sort of… I mean my colleagues could tell me I wasn’t a bad person, but that’s not the same thing [*as hearing it from the HCPC*]*” *(Lucy-06)*.

“*They did write me a letter after the hearing… actually they found me to be very empathetic and very understanding”* *(Sandra-14)*.

However, several participants commented on receiving the final outcome by post which felt impersonal and dismissive:

*“I mean even giving me the positive outcome … that there was no case to answer… there was no warmth or acknowledgement of any emotion”(Amy-03)*.

The interpersonal acts that were reported to occur throughout the FTP process such as dismissing individual circumstances and the perceived lack of empathic communication may give rise to or exacerbate underlying feelings of shame, vulnerability and anxiety. In this theme we have drawn attention to the consequences of the FTP process. In the following theme we synthesise the key suggestions that participants had for improving the process.

#### Theme Three: Navigating road blocks and conceptualising alternatives

Whilst going through the FTP process not all participants internalised feelings of powerlessness and loss. Variation in individual coping strategies provided some participants with the ability to navigate the difficulties of the FTP process. For example, some participants found respite or protection in the limited interpersonal communication they received, or reported being ambivalent about being kept updated:

*“Being ignorant to an extent was bliss.”(Steven-02)*.

*“This is my double-edged sword thing, because part of me wanted to know, because I just thought what on earth is going on – but there was another part of me that didn’t want to know.”(Esther-11)*.

Others avoided personal contact with HCPC, *“because that would prejudice the thing”* *(Lucy-06)* or for fear of providing conflicting evidence that would, *“make me look like I lack integrity”* *(Ethan-16)*. As such, some accepted the power of the HCPC as a legitimate and necessary role in enabling the process to remain objective, particularly in contrast to their employer’s processes:

“*The HCPC were much more objective, independent and dispassionate.*” *(Isabel-08)*.

”*Balanced and sensible…absolutely solid… they were weighing things in the balance.*” *(Lucy-06)*.

When presented with the opportunity to provide their side of the story, typically when proceedings reached the hearings stage, opinions were mixed in terms of experiences. Whilst a highly stressful time in their lives, more than half of the participants found the hearings to be a positive opportunity to have their say; *“(it) was quite empowering… I found it quite cathartic in the end….”* *(Samuel-01)*. Others experienced the hearing as antagonistic and the HCPC representatives were perceived as particularly difficult and like: *“a rottweiler attacking you”* *(Robert-10*). Some participants commented on feeling confused by the hearing process; *“It was confusing to me because I got … in the space of two, three weeks, I got 6 or 7 letters.”* *(Robert-10)*. Several participants discussed not receiving guidance about how to prepare their reflective piece for the hearing; *“I’d asked them what they are looking for, and it was ‘Well we can’t tell you that.’”* *(Samuel-01)*. Others incurred considerable costs to secure legal representation, buying themselves a degree of control in order to voice their story fairly. Others described the costs of attending hearings or training courses:

*“I had to spend my life savings literally on trying to defend myself about £40,000 over the 4-year period.”(Alex-15)*.

*“I was required to go to London for a week… it cost us in excess of £1000 which is a lot of money if you’re not earning…The HCPC offices are in a prime location… you have to stay somewhere that’s not exactly cheap…My order asked for me to do a couple of courses… The only course that I found… was again down in London, it was three days. The course fee was £1500.”(Isabel-08)*.

The majority of those who attended hearings had legal representation which they thought was essential to allow them to both cope with and navigate the legal process. In terms of accessing appropriate sources of support, opinions were equally divided between those who felt that: *“they (HCPC) need to be supportive because I think they’ve got a responsibility to the registrants… you know when you pay all these fees and stuff you expect some support back” (Sophie-12)*, and those who did not see the provision of support as part of the HCPC role: *“It never occurred to me that they would provide support” (Lucy-06).* Whatever their expectations, most participants felt that they did not receive any personal support from the HCPC:

*“You know there should be more back-up from a regulatory body I think, that if you’re going through any kind of issues with your registration that you should be able to get more information, more contact, more support – and I don’t think there’s that.” (Robert-10)*.

All of those who thought that it was not HCPCs role to provide support for registrants suggested that HCPC should signpost to appropriate sources of support: *“They can point maybe to big organisations that are used to dealing with it”* *(Samuel-01)*. Those who thought HCPC should be providing more support themselves made various suggestions:

*“I think if they were to have some sort of counselling advisory service of their own… I am actually here for you, not for HCPC.” (Steven-02)*.

*“Formal support mechanisms have to be put in place to support those going through the process… peer support might be helpful.” (Susan-05)*.

Whilst the HCPC appeared to take a one size fits all approach to cases, several participants thought there should be a review of whether the current approach is appropriate for all cases regardless of their severity. Some participants felt that alternatives such as mediation [9], could be used to make the process more flexible, quicker and less expensive:

*“So they’ve just got to tone down their approach to registrants if it’s not a criminal act… if you’re not being suspended you should not be treated like a criminal.” (Alex-15)*.

*“Perhaps they need to look into their hearing process – are there too many hearings, is it too overburdensome on its members – are there things that they’re classing as worthy of a hearing that shouldn’t be… was my hearing delayed because there was a lot of hearings that were just unnecessary or could have been done a different way.” (Samuel-01)*.

Several participants suggested the need for contextual factors to be considered, particularly those which help provide background to their evidence:

*“They need to consider the context in which mistakes and poor practice happen.” (Susan-05)*.

*“Talking to you about your career, listening to where you’ve been, your journey through the work and what led you to what happened, rather than just looking at the incident in isolation and not taking anything into account.” (Sophie-12)*.

Some participants understood that the HCPC needed to communicate in an impersonal way to maintain objectivity. However they felt that this could be an opportunity for transparency, effective communication and information sharing, rather than as a power move:

*“But as I say, we’re human, I’m human, I need to know what’s going on… The HCPC wants something – it’s got to be in by this date – but they don’t reciprocate by letting you know, and I think that’s one of the big ones. It’s a two-way thing – they want us as registrants to supply them with information – they should give us the courtesy by replying and letting us know as well.” (Donald-13)*.

The majority of participants made suggestions about how the process could be amended although a few thought the process itself did not need changing: *“I don’t think there is anything I would change about the process you know. I think it works”* *(Ethan-16)*. Overall reflecting the experiences of the majority of participants, this theme points to the range of ways in which the FTP process could be reorganised or improved.

## Discussion

Findings from our research chime with previous studies, mostly with research reviewing the process for Doctors, indicating the negative psychological impact that FTP processes can have on an individual’s health and wellbeing including the risk of deteriorating mental health [13, 16-19]. Feeling confused, unsupported, powerless, guilty, anxious and ashamed reflect the experiences of other healthcare professionals going through the investigation process [13, 16, 18-19, 22]. Shame is well-recognised as a concept in healthcare professionals and involves a negative reaction to self-worth, impeding learning [23]. As Biggar et al [6] suggest, there is a need to consider how some of the interpersonal interactions between regulators and those being investigated can be improved so as to better enhance the humane treatment of individuals undergoing investigation. Findings from our study indicate that some if not all of the stress experienced during the FTP process could be alleviated by focussing on the macro level structures and processes at an organisational level. Addressing these issues as part of a procedural review supports the findings of Biggar et al [6] who call upon regulators to improve the level of humanity and transparency with which healthcare professionals are treated whilst being investigated. Specifically, Biggar et al [6] suggest that humane treatment includes fairness, transparency, communication, timeliness and empathic contact. Elevating kindness in the regulatory process and balancing this with accountability has also been a recent topic of discussion at the 2021 International Association of Medical Regulators Authorities (IAMRA) conference [24].

Participants from our study explicitly raised a number of potential ways in which the FTP process could be re-organised to address the current depersonalising aspects of the investigation process. These suggestions include firstly paying greater attention to context in order to more appropriately account for the case. The decisions and actions of healthcare professionals cannot be separated from the context in which they are taken. Assessment of such decisions and actions should acknowledge relevant contextual factors including policies, guidelines and technologies that may have shaped or informed a healthcare professional’s response, alongside what may have been required of them as an appropriate response amidst uncertainty [2], and the impact of systemic contextual issues in the pressured environment of healthcare such as staff shortages. Goodwin [2] suggests that regulatory frameworks could expand their definitions of accountability, making them ‘thicker’ so as not to individualise blame but instead reflect on the collaborative nature of decision-making and possible limits to individual control when providing responsive care. The current ‘measure and manage’ approach to patient safety serves to de-contextualise incident reports. By introducing a constructionist narrative approach to make sense of events, much more could be gained in relation to organisational learning [3].

Mediation as an alternative resolution was suggested by participants of this study as a way of accounting for contextual factors. Evidence elsewhere suggests that this may indeed be more effective and beneficial for healthcare professionals under investigation, particularly in the context of FTP processes [9]. For example, in their study exploring how physicians experience mediation in relation to hospital users’ complaints, Schaad et al [25] suggest that mediation enables sensemaking including the processing of emotions which may in some cases lead to behaviour change. Reviewing cases in a more nuanced way may also prove more effective in terms of both cost and time [26].

Secondly, appropriate support and signposting, including organisational, legal, peer and emotional support was called for. Our findings indicate that support avenues and appropriate signposting are crucial in improving the wellbeing of healthcare professionals during and after the investigation process. Largely because the process is highly stressful and in some cases psychologically traumatising, participants felt that knowing who to go to and where to get this support was both essential to them and would be helpful to future professionals. Whilst different to being under investigation, peer support has been evidenced as helpful in instances where healthcare professionals have experienced medical errors or adverse events [27–30]. Other support avenues could include training line managers who are responsible for supporting individuals undergoing an investigation or period of suspension [20]. Table 5 below summarises these issues and recommendations.

**Table 5:** Summary of issues and recommendations across analytical themes

Issues (themes one and two)	Recommendations (theme three)
Disproportionate, inflexible and punitive nature of the process (theme one)	Consider alternative processes that are less legal and take context into account (e.g. mediation); allow registrants a voice and develop a range of processes that depend on the nature of the complaint to address the disproportionate nature of the one size fits all process.
Perceived assumption of guilty before proven innocent (theme one)	Hold face to face meetings and opportunity for registrant to be heard and provide insights into the context of incident(s). Ensure the maxim innocent until proven guilty is felt and experienced by registrants
Time taken by the investigation and the associated uncertainty; Poor communication about the process and timelines (no road map) (theme one)	Reduce time taken and keep registrants informed at each step of the process to reduce uncertainty; provide clear timeframes to reduce psychological burden on registrants; provide case specific information as well as advise on worst case outcomes; actively provide more guidance to registrants, including what type of evidence is required, appropriate reflective approaches to take and suggested structures.
Lack of compassion and empathy for registrants (theme one)	Ensure HCPC staff themselves feel supported so they in turn they can better support registrants. Increase empathy and staff continuity; train staff to be more responsive, kinder and more compassionate; provide continuity where possible including access to the same case manager/hearing personnel throughout.
Public exposure, shame and associated impact on reputation including impact on employment (theme two)	Ensure the process is confidential and the registrant anonymous until after the hearing unless registrants are suspended; with registrant details not being in the public domain when no case has been proven. Reduce stigma associated with process allowing participants to be more open
High costs and associated financial losses (theme two)	Consider access to free legal support and running panels in more regional locations or via online technology.
No voice in the process for registrants to provide context and be heard (theme two)	Increase opportunities for registrants to be heard using face to face contact to enable HCPC to hear the registrant’s story and more contextual details, particularly at the beginning information gathering stage.
Lack of support (theme two)	Increase support for registrants by signposting and creating support networks including peer support or support line.

Whilst previous research highlighted a number of similar recommendations to those reported here about how to improve the FTP process for healthcare professionals, there is a lack of guidance regarding how to implement changes [31]. The UK GMC have made a number of changes to their process since 2015 including changing the language they use to correspond to registrants and setting out the investigation process more clearly [22]. Future research may now seek to explore in what ways these recommendations can be put into practice at both the level of institutional regulator culture and by frontline employees working at the interpersonal level with those under investigation. Evaluating the impact of these changes, as well as barriers to implementation may also highlight areas of effectiveness, resistance or impracticality.

## Limitations

This study was dependent on registrants volunteering to participate. As such it is possible that this introduced a bias to the sample: with either those who had more negative experiences volunteering, or those with the most negative experiences being unwilling to take part or re-live their experiences. We did however ensure that the experiences of those who volunteered to participate came from a rich and varied sample across professional groups including a variety of experiences of the FTP process.

## Conclusions

Findings highlight the significant psychological impact of the process on individuals; explained by both macro organisational level factors including poor communication; lack of information; and limited support avenues, as well as the micro impact of these structural processes on sense of power and vulnerability of individuals. Key suggestions for improvements to the process include better signposting to appropriate support and greater nuance when dealing with cases including consideration of registrant contextual factors (e.g. through mediation). In the wider context of healthcare workforce shortages, the priority to retain well-motivated and safe staff to enable high quality patient care is critical. Healthcare professional regulators have an important role to play in protecting the public. In all cases, but particularly those cases where there was little or no risk to the public (as was the case for the majority of those we interviewed), some sense of proportionality and the maxim of registrants being innocent until proven guilty is required. Furthermore it is vital that changes are introduced that ensure that both the registrants needs and regulator duty of care to patients are considered and appropriate fair and transparent processes and support are provided.

## Supplementary Information

**Additional file 1.**

**Additional file 2.**

## Data Availability

The datasets generated and/or analysed during the current study are not publicly available due to confidentiality but are available from the corresponding author on reasonable request.

## Abbreviations

CCC: Conduct and Competence Committee
FTP: Fitness to practice
GDPR: General Data Protection Regulation
GMC: General Medical Council
HCPC: Health and Care Professions Council
ICP: Investigating Committee Panel
IAMRA: International Association of Medical Regulators Authorities
PTSD: Post traumatic stress disorder
UK: United Kingdom

## Footnotes

1 This used to include a 16th profession - social work -which was regulated by the HCPC between 2012 and 2019. Social Work England took over from the HCPC as England’s social work regulator on 2nd December 2019. The 15 professions include: Arts therapists; Biomedical scientists; Chiropodists/ podiatrists; Clinical scientists; Dietitians; Hearing aid dispensers; Occupational therapists; Operating department practitioners; Orthoptists; Paramedics; Physiotherapists; Practitioner psychologists; Prosthetists/ orthotists; Radiographers and Speech and language therapists.
