# Supplementary material for: Living life in limbo: experiences of healthcare professionals during the HCPC fitness to practice investigation process in the UK
Source: BMC Health Serv Res. 2021 Aug 19;21:839. doi: 10.1186/s12913-021-06785-7 (PMC8375211; doi:10.1186/s12913-021-06785-7)
Supplement: Supplementary file 1 — Additional file 1. [file 12913_2021_6785_MOESM1_ESM.docx]

Living life in limbo: Experiences of healthcare professionals during the HCPC fitness to practice investigation process in the UK

**Interview topic guide**

**HCPC registrants experience of the fitness to practise process**

Thank you for agreeing to speak with me today about your experiences of going through the fitness to practise process. I work in the Workforce Organisation and Wellbeing team at the University of Surrey.

This research has been commissioned by the Health and Care Professions Council (HCPC) because they are concerned for the wellbeing of their registrants and want to ensure the fitness to practice process is as caring as possible. While we are conducting this research for the HCPC, it is important to say that we are completely independent from the HCPC. We will not tell them that you have taken part in this study.

You have been invited to take part because you have been through the process in the past 12 months. This will form the basis of our discussion today.

Really, I just want to hear your story and your experiences of the process so while I may ask some questions as we go, hopefully they will not interrupt your flow. Before we start, do you have any questions for me?

Great, thanks.

**Background/current occupation**

1. Tell me about your current role…

PROMPTS:

*Current occupation? Length of registration with HCPC?*

*How long have you been a [insert as appropriate]? When did you qualify?*

*Have you always worked in the NHS?*

**Fitness to practice – your story**

1. Tell me your fitness to practise story, starting wherever you wish…..

PROMPTS:

*When did you become aware that there was a problem?*

*Were you referred or did you self-refer?*

*What was/Was there an event that led to the referral?*

*How were you notified that you had been referred?*

*Had you been through a disciplinary process at work which triggered this referral?*

*What was your first reaction; can you remember how it felt?*

1. How well informed did you feel throughout the process?

PROMPTS:

*Was it clear who you could talk to and how to do that?(e.g., single point of contact)*

*Did you find it difficult or awkward to speak with relevant staff?*

*What types of issues did you ask about?*

*Did written communications make sense to you?*

*Were you told about your rights in terms of legal support?*

*Did you contact any regulatory bodies (beyond the HCPC) for help or support?*

*Were there particular areas where you could have done with more/less information?*

*Was there ever contradictory information given to you?*

*Were you clear about progress and what would happen at each stage?*

*Do people in situations like this want all of the information – good and bad?*

1. How engaged you were in the process?

PROMPTS:

*Are there any things in which you would have liked more say?*

*Did you feel you had the opportunity to provide your feedback (thoughts/comments)?*

*Were you part of the decision-making process?*

*Did you feel that your voice was heard?*

*Did you feel listened to?*

1. Your relationships with the staff you met

PROMPS:

*How many staff did you deal with through the process?*

*Are there any meetings or interactions that particularly stand out to you?*

*What was your experience of support in general?*

*Were there any experiences with staff that prevented or deterred you from raising an issue?*

*How far would you say you have been treated with respect, courtesy and sensitivity?*

1. Tell me about the experience of the investigation process (if not already covered) and the panel hearing (if applicable)…

PROMPTS:

*What were the emotional highs and lows of the investigation process?*

*Can you remember how you felt before you went into the hearing [if invited to panel hearing]?*

*How did you experience the hearing? The staff on the panel?*

*What was the tone of the hearing like?*

*Did you feel listened to?*

*What processes could HCPC do differently that would make the experience better for registrants?*

1. What other types of support did you have?

PROMPTS:

*Professional: Did you have any support from professional groups? To what extent have they been able to play a positive role in helping you deal with the experience? (e.g., community, belongingness/acceptance, information, practical help). Were there any downsides?*

*Partner, family, friends, fellow workers, peers, etc.: as above.*

*Fellow registrants: as above. Would it have helped to be able to speak with registrants who had been through the process before you?*

*What different support could HCPC provide/what things could be done differently that would make the process better for registrants?*

1. How did you cope throughout the process?

PROMPTS:

*What effects has the experience of going through this process had on your health? (your mood/mental health, behaviour, physical health)*

*What advice would you give to other people about how to cope with this process?*

**Overall: Best and worst bits of the FTP process**

1. Where would you say are the most crucial points in the fitness to practice process? Moments of truth? The emotional highs and lows for you…
2. What were the worst and best bits of your experience of the process?
3. Crucial touch points? The parts that should be focussed on in the design of this process?
4. If you were able to change or improve three aspects of the process, what would they be; what would you would do?
5. Is there anything else you would to share with me today?

Thank you for sharing your experiences with me today. It’s been really interesting to hear your story and to understand more about your experiences of going through the fitness to practise process. I’m aware that this discussion might have raised some difficult emotions so I just want to check that you’re feeling okay at the moment? *[if not, discuss debriefing sheet with signposting to support mechanisms]*

In terms of what happens next, we are speaking with other HCPC registrants about their experiences. We then intend to develop a script for a film for the HCPC using anonymised quotes from these interviews. This will help the HCPC to understand what it is like to go through the fitness to practise process, what support people need, and how the process might be improved. We will also make recommendations to the HCPC in a written report and will include anonymised quotes from the interviews in this (again, you will not be personally identifiable). Finally, we will create a publication to be published in the academic literature so that the results of this study can be understood more widely. We again will use anonymised quotes from the interviews conducted.

Does that all sound okay to you? Would you like to review the film script? *[for assurance of anonymity being maintained]*

Do you have anything else you would like to ask me?

**Thanks again for taking the time to speak with me today.**
