# Supplementary material for: Living life in limbo: experiences of healthcare professionals during the HCPC fitness to practice investigation process in the UK
Source: BMC Health Serv Res. 2021 Aug 19;21:839. doi: 10.1186/s12913-021-06785-7 (PMC8375211; doi:10.1186/s12913-021-06785-7)
Supplement: Supplementary file 2 — Additional file 2. [file 12913_2021_6785_MOESM2_ESM.docx]

Living life in limbo: Experiences of healthcare professionals during the HCPC fitness to practice investigation process in the UK

**Final coding structure for analysis of HCPC interview data**

| **Category** | **Analysis code** | **Description** |
| --- | --- | --- |
| **1. Participants and interview** | | |
| **Experience of interview** | **Motivation to participate** | Tell story, improve for others |
|  | **Impact of interview** | Closure, cathartic, distress |
| **2. Context** | | |
| **Experience of work** | **Feelings about workplace** | Love job, sad about conflict values |
|  | **Motivation to work** | Always done job, client focus, good at job, innovator, respected |
|  | **Commitment to work** | Conscientious |
| **Workplace culture and relationships** | **Workplace relationships** | Abusive, unsupportive, misrepresentation at HCPC, supported, good relationships |
|  | **Workplace pressure** | Workload, organisational change, professional expectations, stressful |
|  | **Workplace culture** | Fear of failure, poor policy, no emotional support, participant raise issues, value clash |
|  | **Workplace impact** | Distress, anger, stress, sickness, depression, burnout |
| **Prior health + life issues** | **Life issues** | Move house, masters |
|  | **Prior physical health** | Exhaustion, epilepsy, chronic pain, gynae |
|  | **Prior mental health** | Anxiety, stress, depression, ADHD, dyslexia |
| **3. FTP process** | | |
| **Precipitating events** | **Description of precipitating event** |  |
|  | **Employer actions** | Suspend, investigate, disciplinary, capability, warning, demote, dismiss, reinstate |
|  | **Impact of precipitating event** | Shock, distress, anxiety, depression, anger, off sick, humiliation, isolation, acceptance, resign from work, practical issues |
| **4. Participant experience of FTP**  **4.1 Description of process** | | |
| **Description of overall experience** | **Overall description of process** | Horrific, dehumanising, horrendous, nightmare, interesting, satisfactory |
|  | **Variation in experience** | Investigation-v-hearing |
|  | **Low points** | **Investigation** - public exposure, remote, criminalised, receive paperwork / **Whole process** / **Referral** - paperwork, delay response, notification that going to full investigation / **Hearing** |
|  | **High points** | **Hearing** **-** validation/ **Outcome** - validation / **None** / **Ending** / **Progress** - hearing date |
|  | **Overall opinion about HCPC** | Objective, sane, bastards, independent, dispassionate, hypocritical, unprofessional, effective, unable to challenge, fear of consequences, aim to strike off, contrast HCPC behaviour and expectation of registrants |
| **4.2 Complaints about process** | | |
| **Nature of process** | **Process inappropriate** | Legal, punitive, formal, inflexible, disproportionate, unfamiliar |
|  | **Process efficiency** | Cost, not efficient |
|  | **Process ineffective** | Consider context, safeguarding issues |
|  | **Process unfair** | Criminalised, assumption of guilt, engage with or believe complainant, biased, not acknowledge actions taken by registrant (e.g. self-referral) |
|  | **Time taken** | Duration, drags on, postponements, allow employer slow response, provides time to reflect, recover |
|  | **Public exposure** | Website - delays removing, triumphalist, no review of transcript, inaccurate reporting of referral / Informing others - need to inform / Post - others access/paperwork registrant named |
| **Communication content** | **Process not explained** | Process and timelines unclear, clear flow charts, not case specific |
| **Communication sensitivity** | **Sensitivity** | Dehumanising, punitive, not equal, neutral, cold, warm, empathic, expect impersonal, not take participant schedule into account, compassion |
| **4.3 Outcomes** | | |
| **Life impact** | **Losses** | Job, business, income, house, profession, friends, respect, reputation, pride, status, everything, control, loss of registrants in profession |
|  | **Impact on working practices or relationships** | Vigilant, lack of trust, relationships, ostracised, no reconciliation |
|  | **Impact on life decisions** | Life decisions - delay child |
|  | **Cost of process for participants** | Attend hearing, job loss, lost income, comply with conditions, legal costs, time to prepare hearing |
|  | **Uncertainty** | Career, livelihood, house, family, in limbo, can't plan or apply for / change jobs |
|  | **Impact on physical health** | Seizures, psoriasis, sleep, cardiac, drinking, weight |
| **Wellbeing impact** | **Impact on wellbeing of registrant and others** | Shock, anxiety, PTSD, stress, fear, anger, constant worry, isolated, cathartic, disbelief, identity/ Impact on others - family, foster carer |
|  | **Shame** | Shame and guilt |
|  | **Anything gained** | Life decisions (career choice, health, house, wife, child, masters), Personal development (perspective, assertiveness, approach to work, insight, reflection, resilience, knowledge, skills, patience) |
| **Participant response** | **Approach to process** | Open, honest, personal development, avoidance, reflection, challenge, research, determination, reasons for gains (positive approach, personal effort) |
|  | **Individual coping strategies** | Reflection, Research, Determination, Resilience, Distraction |
| **4.4 Experience of referral** | | |
| **Description of referral experience** | **Description of referral experience** | Referred by (self, employer, service user), no warning |
| **Impact of referral** | **Impact of referral** | Breakdown, seizures, fear, distress, shock, depression, suicidal, off sick, anger, horrified, stress |
|  | **Attitude to referral** | Acceptance, wrongly accused, harsh, welcome |
| **Complaints about referral process** | **Referral communication** | Delays in information after referral |
|  | **Referral paperwork** | Volume, confusing, accuracy - redacted mitigating circumstances, confidentiality, not anonymous |
| **4.5 Experience of investigation** | | |
| **Description of investigation experience** | **Description of investigation experience** | Horrific, horrendous, waiting |
|  | **Investigation low points** | Public exposure, remote, criminalised, notification of full investigation, receive paperwork |
| **Impact of investigation** | **Impact of investigation** | **Impact** – breakdown, in limbo, isolated, fear, constant worry, anxiety, paranoia, frustration, uncertainty |
| **Complaints about investigation process – communication method** | **Communication method and responsiveness** | No face to face, email fine, prefer post, restrictions on contact by legal advisor restrict HCPC or participant reluctant, changed contact point of contact and teams, different people, consistent, had to chase , not available, not respond, respond to solicitor |
| **Complaints about investigation process – communication content** | **Inadequate information during investigation** | No progress update, unclear what will happen, unclear allegations and consequences, avoidance (ignorance bliss), inadequate course recommendation, inaccurate advice to tell colleagues |
|  | **Nature of communications** | Legal, unclear |
| **Complaints about investigation process – communication sensitivity** | **Lack of sensitivity during investigation** | Pleasant, cold, formal, not consider as person, variation, external legal advisor good |
|  | **Participant voice in investigation** | no opportunity to put my side, unable to challenge, ignore participant request about timescales, legal advisor restricted challenge, able to challenge when external solicitor took over |
| **4.6 Experience of hearing** | | |
| **Description of hearing experience** | **Description of hearing experience** | horrible, horrific, amicable, intense, challenging |
|  | **Variation between hearings** | interim-v-final, health v conduct, between panels |
|  | **Hearing low points** | Receive paperwork, breakdown |
|  | **Hearing / outcome highs** | Making progress, outcome |
| **Hearing impact** | **Hearing / outcome impact** | empowering, cathartic, relief, traumatic, physically sick, distress, anxiety, insight |
|  | **Validation** | Validation |
|  | **Attitude to outcome** | COP reasonable or difficult, suspension difficult, relief, not listen to request to be removed from register, actions not acknowledged |
| **Nature of hearing** | **Complaints about nature of hearing** | Legal, formal, rigid, unfamiliar, like court, biased, unfair, punitive, aiming to strike off |
|  | **Complaints about public exposure at hearing** | Public attend |
|  | **Complaints about short notice of hearing date or appeal deadline** | No time to prepare, ability to appeal, duration of hearing |
|  | **Hearing effective** | Consider context |
|  | **Hearing fair** | Unbiased |
|  | **Hearing facilities good** | Room, refreshments |
| **Hearing communication** | **Complaints about provision of guidance** | Unclear requirements, submission instructions |
|  | **Guidance clear** | Guidance clear in letters |
|  | **Lack of sensitivity at hearing** | Challenging, HCPC rep aggressive not respectful, panel members changed. foreboding, judgemental |
|  | **Participant had no voice at hearing** | Unable to challenge witness, difficult to get point across, not heard, actions not acknowledged |
|  | **Complaints about outcome communication** | By post |
|  | **Participant had voice at hearing** | Opportunity to put my side, panel listened |
|  | **Empathy at hearing** | Friendly, clerk and independent legal advisor empathic, panel respectful supportive |
| **5. Support** | | |
| **Support from HCPC** | **Expectation of support from HCPC** | Not their role to support, should provide information on process, progress, guidance and signpost, responsibility to registrant expect representation more for money paid |
|  | **Provision of support by HCPC** | Not much, not helpful, clerk supportive at hearing, aim to strike off and attack not support, ineffective signpost |
| **Professional support** | **Legal support** | Required, helpful, undermine or support faith in HCPC |
|  | **Union Professional body, Conflict resolution specialist** | - not great, brilliant, unsupportive, advised to admit |
|  | **Medical** | GP supportive, counselling helpful, crisis team mental health PTSD |
| **Other support** | **Friends and family** |  |
|  | **Colleagues and other registrants** |  |
| **Support barriers** | **Practical barriers to obtaining support** | Professional membership registration, Cost, How to identify, Short notice |
|  | **Attitudes preventing use of support** | Ability to represent effectively, Burden to others |
| **6. Improvement recommendations** | | |
| **Improve process** | **Change nature of process** | Less legal, preliminary hearings, face-to-face meetings, same panel, flexibility (alternative process, separate for non-contested), timeline (reduce time taken, set limits), confidentiality (not on website or named in documents until decision) |
|  | **Improve fairness** | Consider context, be consistent, no assume guilt, review professional standards |
|  | **Reduce cost to participants** | Less expensive process, cost for registrants (regional location, technology, later times) |
| **Improve communications** | **Improve nature of communications** | Less foreboding, clear, simplify communications |
|  | **Improve provision of information** | Notify about referral, clarify processes, case specific outcomes, accurate timelines, clarify allegations, communicate progress |
|  | **Improve provision of guidance** | Clarify what required, provide accurate guidance |
|  | **Improve sensitivity** | Empathy, recognise emotion**,** compassion, warmth, humanity, respect, mindful of impact |
|  | **Ensure anonymity for registrants** | Registrants anonymous, information on website |
|  | **Improve responsiveness** | timely response, point of contact (consistent, internal coordination) |
| **Improve provision of support** | **Signpost to sources of support** | Main organisations, registrants with experience of process |
|  | **Provide emotional support** | **S**upport network, peer support / **Recovery** - compensation**,** reconciliation **/ Provide support -** for those not in union, create champions in profession |
|  | **Advice for other registrants** | **Advice re coping strategies -** Support (union, legal, family, friends, employers) Approach (open, honest, integrity, self-care, communicate, be strong, challenge HCPC positive, maintain hope, comply with process, be patient) |
